# Cardiac lipoma in a young lady

**DOI:** 10.1002/ccr3.8299

**Published:** 2023-12-20

**Authors:** Azin Alizadehasl, Mina Mohseni, Alia Bahramnejad, Kamran Roudini, Maryam Mohseni‐Salehi, Maryam Favaedi, Hoda Hakimian, Amir Dousti

**Affiliations:** ^1^ Cardio‐Oncology Research Center, Rajaie Cardiovascular Medical and Research Center Iran University of Medical Sciences Tehran Iran; ^2^ Echocardiography Research Center, Rajaie Cardiovascular Medical and Research Center Iran University of Medical Sciences Tehran Iran; ^3^ Cancer Institute, Imam Khomeini Medical Center Tehran University of Medical Sciences Tehran Iran; ^4^ Congenital Heart Diseases Research Center, Rajaie Cardiovascular Medical and Research Center Iran University of Medical Sciences Tehran Iran; ^5^ Interventional Cardiology Research Center, Rajaie Cardiovascular Medical and Research Center Iran University of Medical Sciences Tehran Iran

**Keywords:** cardiac tumor, cardio‐oncology

## Abstract

**Key Clinical Message:**

To confirm the diagnosis of cardiac lipomas, it is crucial to use multimodality imaging and also histopathology examination if the patient underwent surgery. But surgery is not needed in many cases unless there are life‐threatening situations.

**Abstract:**

Cardiac lipoma is a rare condition which is believed as a benign tumor; here, we want to present a case of young adult lady who came to our hospital complaining of chest pain and diagnosed cardiac mass by echocardiography that underwent cardiac MRI which showed cardiac lipoma and managed conservatively by serial echocardiography.

## 1 INTRODUCTION

Primary cardiac tumors are rare. The overall prevalence of primary cardiac tumors is 0.001%–0.03% on the basis of epidemiologic studies and autopsy series which 84.6% of these are benign.^1^

Cardiac lipomas account for about 10% of all primary cardiac tumors. Lipomas are composed of mature adipocytes and can form in any part of the heart. Lipomas are encapsulated and well surrounded tumors, and often, they are benign and slow growing. Most lipomas occur in the right atrium or left ventricle. Lipomas originating from the sub‐endocardium can cause obstruction, and those originating from the myocardium can cause arrhythmias. In addition, in sub‐epicardial cases, they can cause pressure on the coronary arteries or the pericardial space. Cardiac lipomas are usually asymptomatic and are therefore often found incidentally. In symptomatic cases, the patient's symptoms depend on the location of the mass and obstruction in the cavities or valves of the heart.^2^, ^3^

## 2 CASE PRESENTATION

**FIGURE 1:**
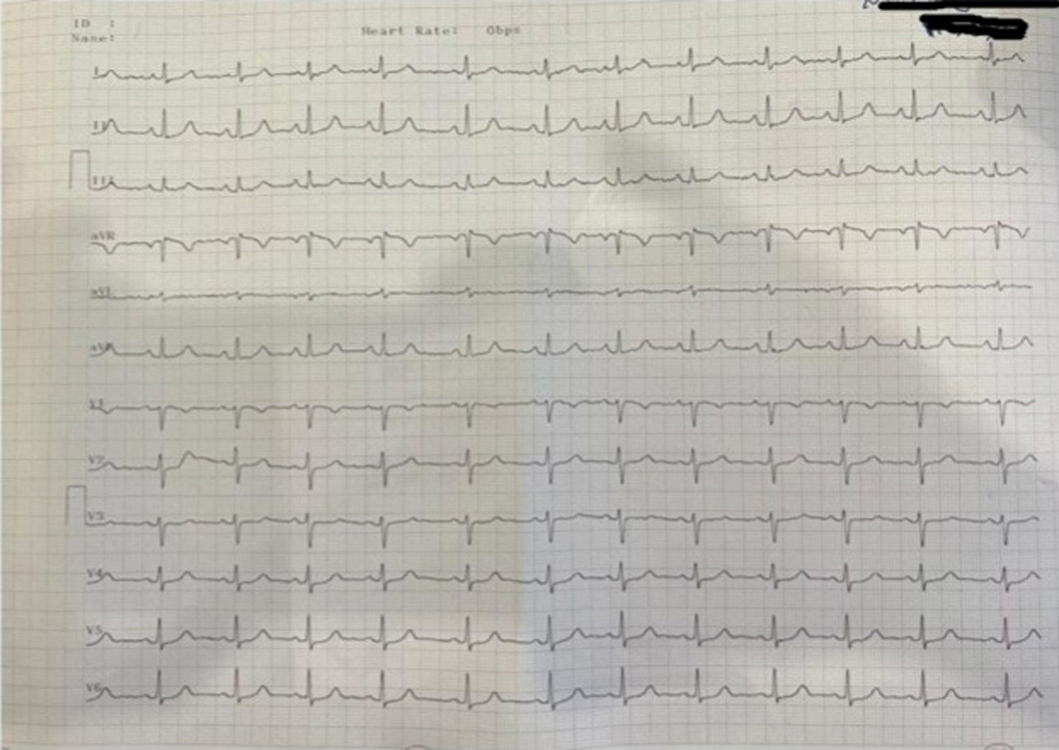
Electrocardiography (ECG).

A 22‐year‐old woman with a history of type1 diabetes referred to the emergency room complaining of atypical chest pain and palpitation. Electrocardiography (ECG) was unremarkable with no arrhythmia (Figure 1).

**FIGURE 2:**
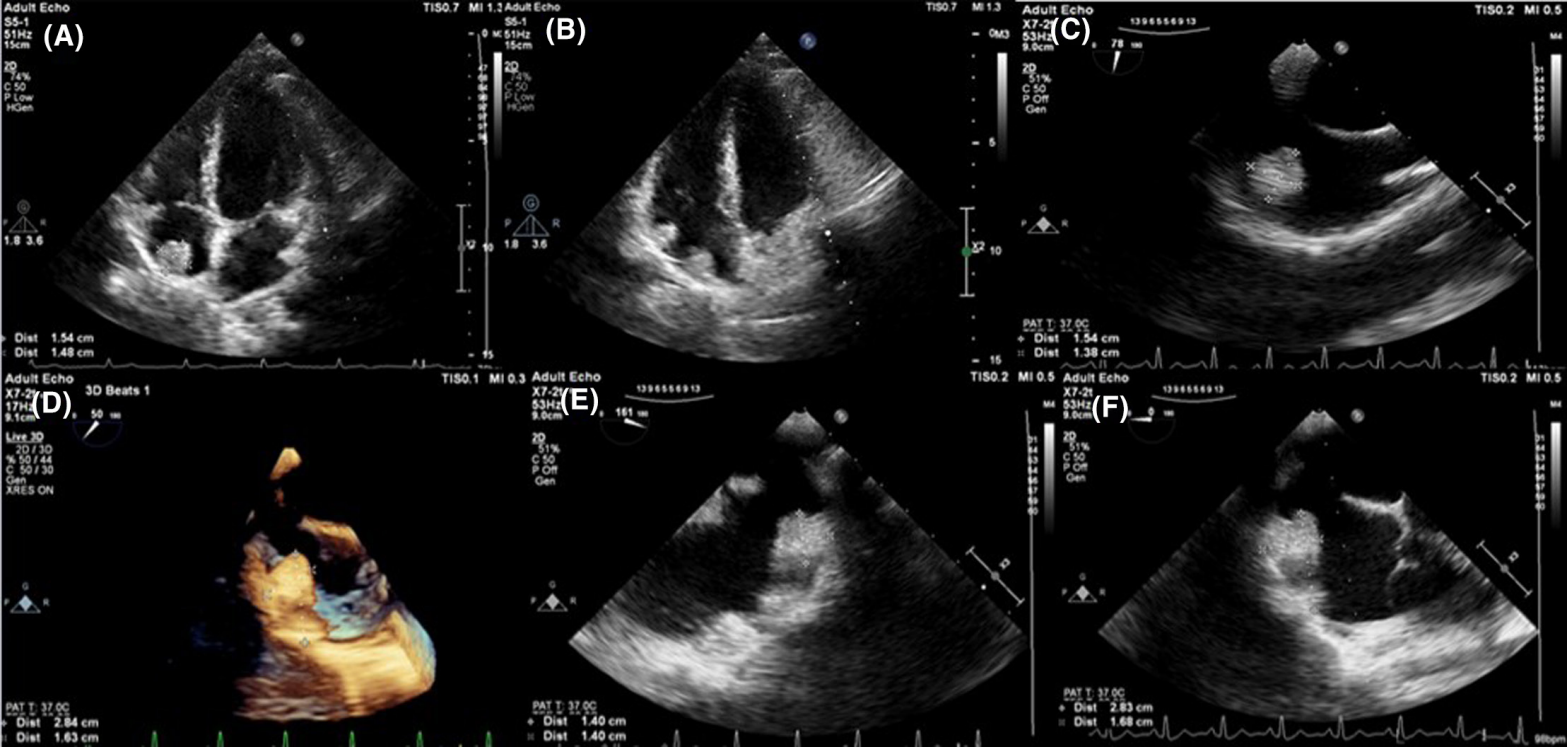
(A and B) Transthoracic echocardiography, (C–F) transesophageal echocardiography.

On transthoracic and transesophageal echocardiography, the size and function of the right and left ventricles were normal, the size of the atria was normal, and there was mild regurgitation of the mitral and tricuspid valves. TRG was 23 mmHg, and PAP was 28 mmHg. There is homogenous fixed bilobated mass attached to RA roof (size = 2.8 × 1.7 cm), no mass in other valves or cardiac chambers. (Figure 2).

**FIGURE 3:**
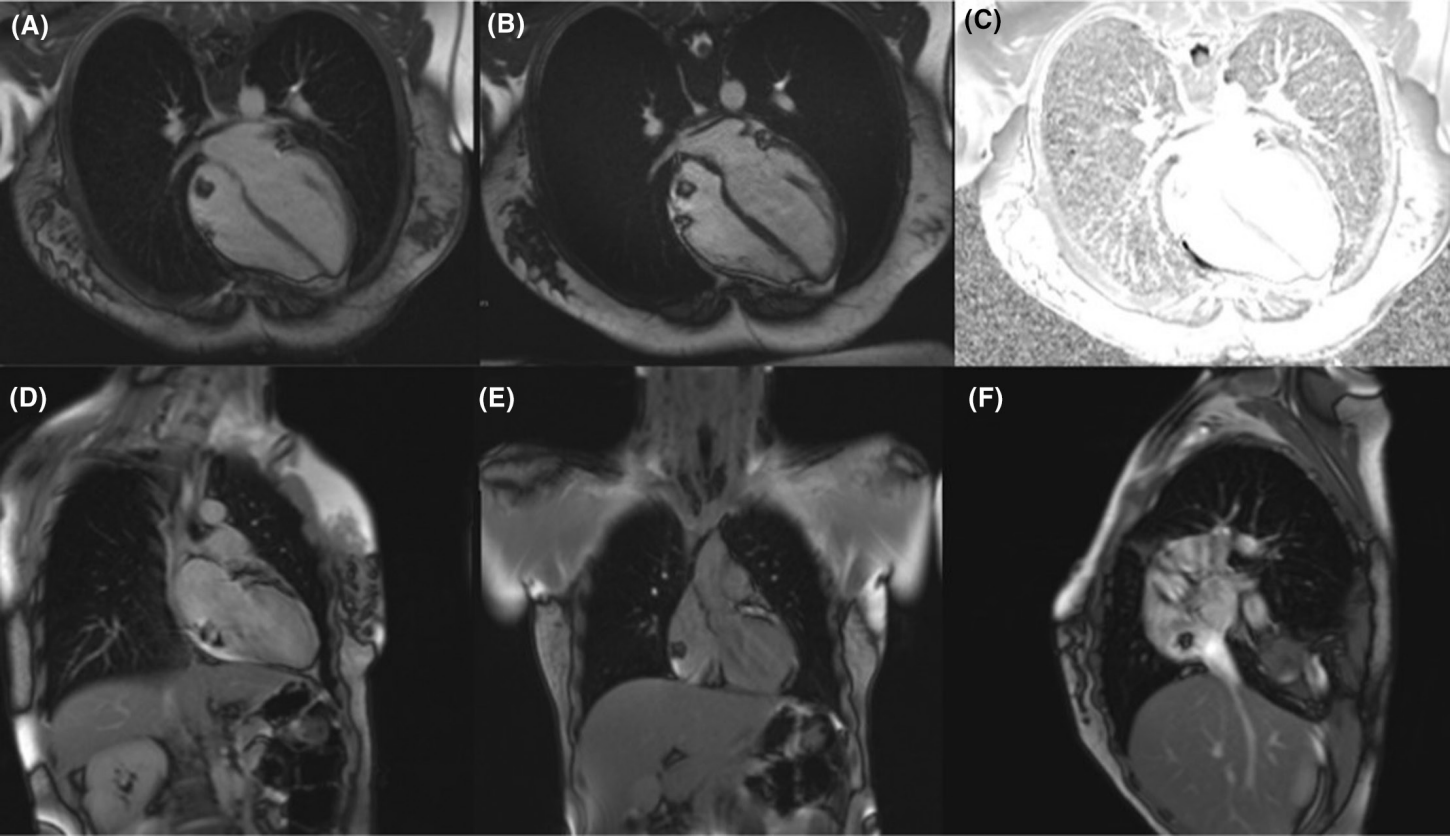
Cardiac magnetic resonance imaging.

Electrocardiogram and CXR were unremarkable. She underwent cardiac magnetic resonance imaging (CMR). Her CMR showed a round well‐defined mass attached to postero‐superior RA wall with a stalk measured 10 x 11 mm without evidence of hemodynamic obstruction or compression. In the STIR/T2 weighted‐sequences, the mass is low signal, and in the T1 weighted‐sequences with fat suppression images, the mass is low signal. In the first pass perfusion sequence, the mass has negligible perfusion. In the early‐enhancement imaging, the mass has no enhancement, and in the late‐enhancement‐sequences, the mass has negligible enhancement. Due to MRI tissue characterization criteria, cardiac lipoma is the most likely diagnosis. As a result, we decided to observe the patient with close follow‐up and not perform surgery on the patient. (Figure 3).

**FIGURE 4:**
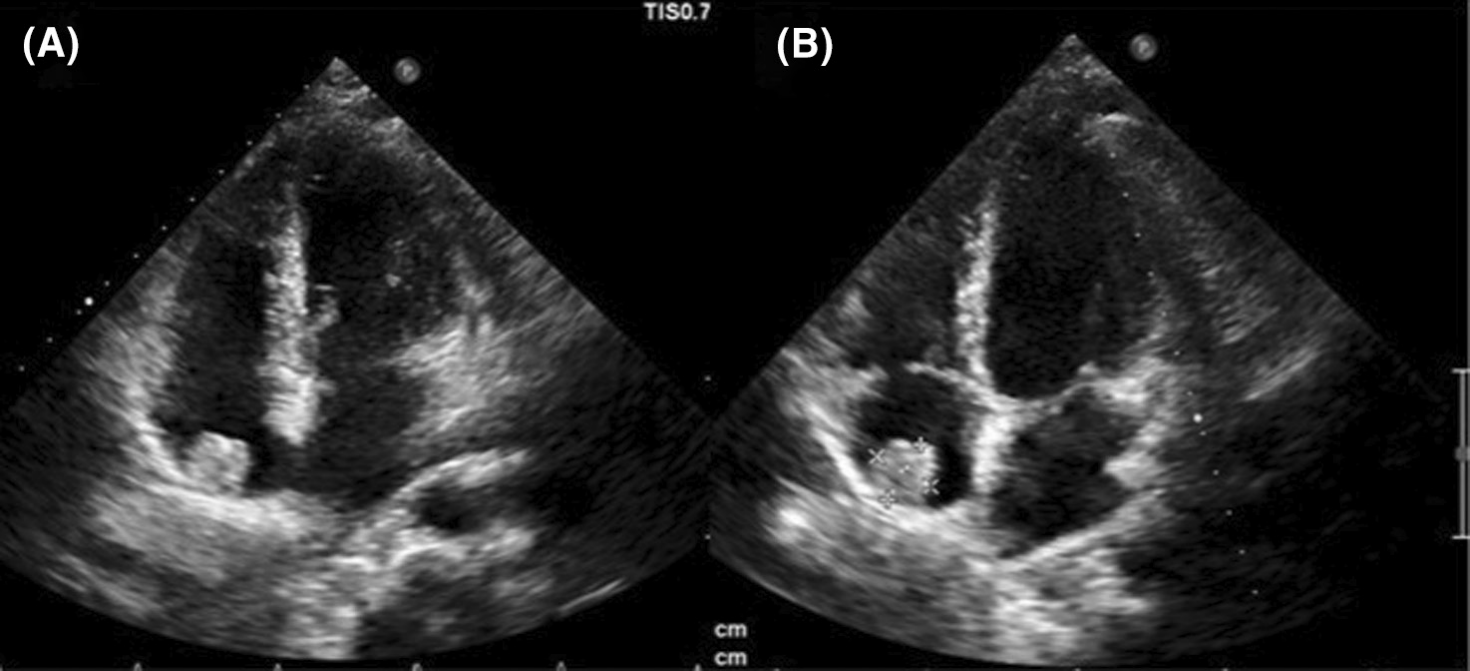
Follow‐up transthoracic echocardiography.

In the follow‐up echocardiography 6 months later, no change in the size of the mass was observed and the patient was completely asymptomatic. (Figure 4).

## 3 DISCUSSION

Echocardiography is an available and suitable tool for the initial evaluation of the mass and checking the presence of obstruction in the intracardiac cavities or heart valves. Lipoma in echocardiography usually presents as a broad‐based, homogenous, encapsulated and echogenic mass, usually without calcification within the cardiac chambers or the pericardium with a clear boundary. Lipoma exhibits no enhancement in echocardiographic contrast imaging.^4^, ^5^, ^6^

CT scan of the heart and cardiac magnetic resonance imaging (CMR) are very useful for more accurate diagnosis. Most lipomas in cardiac magnetic resonance imaging (CMR) are homogeneous fat signal on all sequences. To confirm the presence of fat in cardiac tumor, signal drop out on fat‐suppressed sequences can be used. Lipoma is a hypovascular non‐enhancing tumor and sometimes may demonstrate septa.^7^, ^8^

Its treatment is resection in symptomatic cases, but in asymptomatic cases, the patient can be monitored without resection with serial imaging. If resection is performed, recurrence is rare, but close follow‐up is still recommended.^9^

## AUTHOR CONTRIBUTIONS

**Azin Alizadehasl:** Methodology; project administration; supervision. **Mina Mohseni:** Validation. **Alia Bahramnejad:** Methodology; validation. **Kamran Roudini:** Methodology; project administration; supervision. **Maryam Mohseni‐Salehi:** Resources. **Maryam Favaedi:** Resources. **Hoda Hakimian:** Project administration; writing – original draft. **Amir Dousti:** Methodology; writing – review and editing.

## FUNDING INFORMATION

There was no funding.

## CONFLICT OF INTEREST STATEMENT

The authors have nothing to declare, there was no conflict of interest.

## CONSENT

Written informed consent was obtained from the patient to publish this report in accordance with the journal's patient consent policy.

## Data Availability

All data are available at Rajaie cardiovascular medical and research center and are accessible via e‐mailing the corresponding author.
